# Innovative Solutions for Obesity: Evaluating gastric bypass stents in Postlaparoscopic Sleeve Gastrectomy Weight Management

**DOI:** 10.1055/a-2936-2289

**Published:** 2026-08-28

**Authors:** Zhengqi Li, Yuntao Nie, Pengpeng Wang, Baoyin Liu, Biao Zhou, Nianrong Zhang, Siqi Wang, Sai Chou, Zhe Wang, Hua Meng

**Affiliations:** 1 Department of General Surgery 36635 China-Japan Friendship HospitalBeijingBeijingChina; 2 Department of General Surgery & Obesity and Metabolic Disease Center 36635 China-Japan Friendship HospitalBeijingBeijingChina

**Keywords:** other focus (of reviewers), GI surgery, endoscopy upper GI tract, dilation, injection, stenting, endoscopy lower GI tract, stenting

## Abstract

**Objectives** The incidence of regaining weight after laparoscopic sleeve gastrectomy (LSG) is high. TONGEE duodenum-jejunal bypass liner (DJBL) has been proven to reduce the weight of obese patients. The present study aims to investigate the effect of TONGEE DJBL on weight loss in patients who have regained weight after LSG.

**Methods** A cohort study with two nonrandomized groups was conducted at the China-Japan Friendship Hospital in Beijing. The recruitment period was from August 2023 to September 2025 for patients with simple obesity undergoing DJBL treatment and those who had regained weight after LSG surgery. We then compared the weight loss effects of the two groups using univariate and multivariate analyses.

**Results** A total of 30 patients with simple obesity and 16 patients who had regained weight after LSG surgery were recruited. The incidence of complications in the two groups, total weight loss percentage (10.98 ± 5.96 vs10.36± 3.78, 95% CI: 8.84, 13.11), and excess weight loss percentage (50.87 ± 37.28 vs 50.38 ± 29.16, 95% CI: 37.54, 64.22) do not have statistical difference ( *P* > 0.05). Three months after TONGEE DJBL surgery, both groups of patients showed a decrease in body fat percentage and an increase in muscle percentage.

**Conclusions** TONGEE DJBL can be used as an endoscopic treatment option for patients who regain weight after LSG surgery.

## Introduction

In recent years, laparoscopic sleeve gastrectomy (LSG), due to its relatively simple operation and fewer complications, has far surpassed another classical surgical method, Roux-en-Y gastric bypass (RYGB), becoming the most performed bariatric surgery in China, accounting for 89.4%. 1 2 However, the follow-up results after LSG showed that the weight recurrence rate was 5.7% at 2 years after surgery, and the regain rate was usually 19.2%~75.6% after more than 5 years after surgery. 3 4 Studies have shown that 33% of patients undergo revision surgery at 10 years after LSG, and about 66% of the revision procedures are due to regain of weight. 5

The duodenal-jejunal bypass liner (DJBL) is an endoscopic weight loss treatment that replicates aspects of gastric bypass surgery in a minimally invasive way. Its mechanism involves placing a 60-cm impermeable sleeve endoscopically via the stomach. This liner extends into the duodenum and upper jejunum, physically separating ingested food from biliopancreatic secretions within that segment of the small intestine. This separation reduces digestion and nutrient absorption, leading to weight loss and metabolic improvements.

EndoBarrier, the most widely studied DJBL device in the United States, has demonstrated efficacy in treating severe obesity and improving type 2 diabetes mellitus in clinical trials. 6 7 8 However, adverse events, particularly liver abscesses, 9 10 have limited its widespread adoption.

Compared to Endobarrier, TONGEE DJBL has several technical modifications. Firstly, the improved sleeve-shaped biomaterial provides better barrier performance, reducing the growth of harmful bacteria that may cause liver abscess. Secondly, the bracket spikes on the anchoring system of the device have been modified to avoid damage to the duodenum. Thirdly, the improved conveying and recycling system can eliminate the need for perspective guidance during the insertion and removal of the DJBL system. 11 These innovative aspects have helped it obtain the breakthrough device design from the US Food and Drug Administration (ID: Q242030).

The present study investigates a novel duodenal-jejunal bypass device: a gastric bypass stent (Tangji Medical, Hangzhou, China) to treat obesity and evaluate its effectiveness and safety in controlling weight and obesity-related metabolic indicators.

## Methods

### Study Design

This cohort study with two nonrandomized groups was designed to assess the feasibility and safety of the TONGEE DJBL used for the treatment of obesity and weight recurrence after LSG.

### Patients

Simple obese patients and patients who regained weight after LSG were enrolled in this study. The study was conducted at the General Surgery & Obesity and Metabolic Disease Center, China-Japan Friendship Hospital, from August 2023 to September 2025. This study complied with the Declaration of Helsinki and was approved by the ethics committee of our hospital (approval number 2023-KY-125). Written informed consent was obtained from all the patients upon inclusion in the study. The study was registered at clinicaltrials.gov (NCT06801496).

### Inclusion and Exclusion Criteria

The inclusion criteria are as follows:

1. Patients who were diagnosed with simple obesity or who have undergone LSG;
2. A BMI ≥25 kg/m ^2^
3. Age range from 16 to 65 years;
4. Lifestyle or exercise interventions have been ineffective;
5. Patients who have signed an informed consent form;
6. Patients who had undergone LSG and having a postoperative period of more than 1 year, and any of the following criteria:
  1. BMI increases ≥5 kg/m ^2^ ;
  2. Weight gain ≥10 kg;
  3. A postoperative weight that has rebounded by more than 15% from its minimum body weight;

The exclusion criteria are as follows:

1. Gastroscopy examination and surgical contraindications, including:
  1. Heart failure;
  2. Individuals with persistent breathing difficulties;
  3. Patients with retropharyngeal abscess, severe spinal deformity, or aortic aneurysm;
  4. Unable to tolerate endoscopic examination due to physical weakness;
  5. Acute stage of upper gastrointestinal corrosive inflammation or suspected upper gastrointestinal perforation;
  6. Individuals with a large amount of ascites, severe abdominal distension, or severe esophageal varices;
  7. Pregnant women;
  8. Individuals with abnormal bleeding and coagulation functions;
2. Individuals with a history of botulinum toxin treatment within the past 3 months;
3. Endoscopic detection of tumors, active peptic ulcers, etc.;
4. Planned pregnancy within the past 6 months.

### Device

**Fig. 1:**
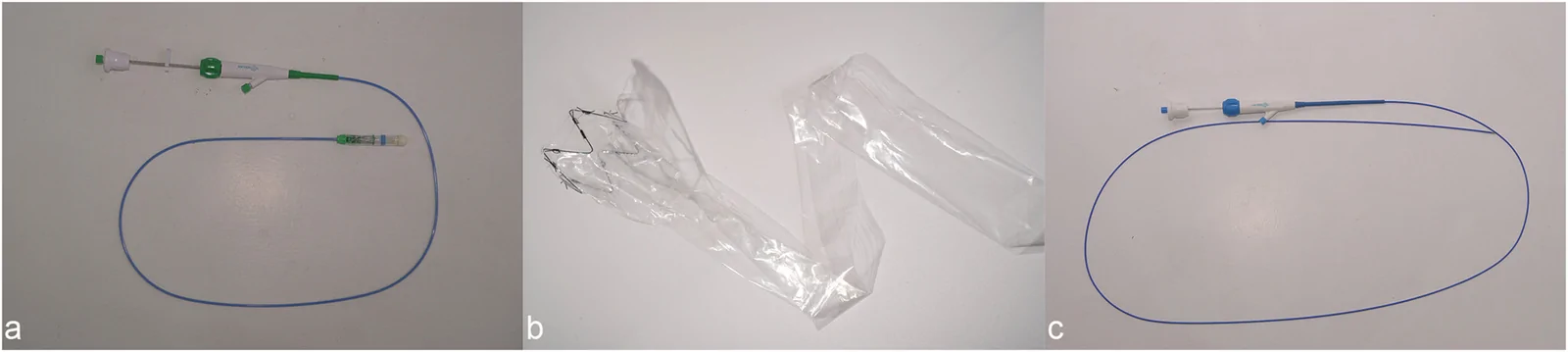
The details of TONGEE DJBL. The TONGEE DJBL system is a disposable endoscopic implant consisting of a delivery system ( **a** ), a sleeve ( **b** ), and a retrieval system ( **c** ).

Fig. 1 shows the details of TONGEE DJBL. The TONGEE DJBL system is a disposable endoscopic implant consisting of a delivery system ( Fig. 1a ), a sleeve ( Fig. 1b ), and a retrieval system ( Fig. 1c ).

### Procedure

**Fig. 2:**
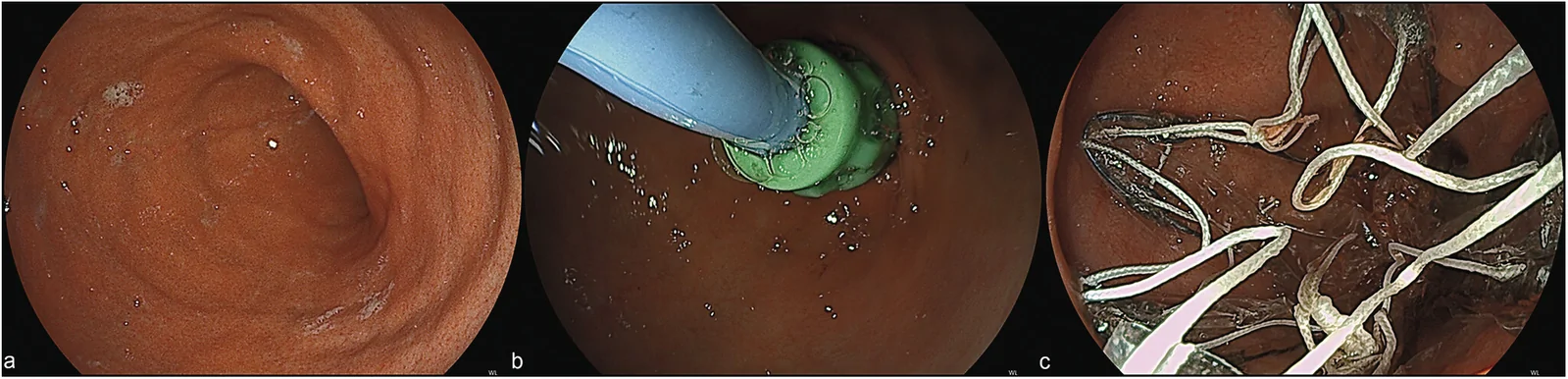
The placement process of TONGEE DJBL in weight recurrence patients after laparoscopic sleeve gastrectomy. The scar can be seen after sleeve gastrectomy ( **a** ). The delivery system was inserted into the introducer sheath ( **b** ). The stent was then deployed successfully upon release ( **c** ).

Informed consent was obtained from all the patients prior to the procedure. Under gastroscopic guidance, the TONGEE DJBL was inserted into the duodenum ( Fig. 2 ). The entire process was performed without X-ray fluoroscopy. On the second postoperative day, upper gastrointestinal imaging was performed to evaluate stent placement. The TONGEE DJBL remained implanted for 3 months. Following implantation, participants initially received a liquid diet for 2 weeks, progressed to a semiliquid diet for the next 2 weeks, with no dietary restrictions thereafter. During stent implantation, the use of other weight loss medications was prohibited. Follow-up examinations were conducted at baseline and 3 months post-implantation. The specific time point is that the patient undergoes the stent removal procedure 3 months after the stent was placed. The method for body composition analysis involves using a bioelectrical impedance analysis device to measure body composition in the morning, while fasting and avoiding water intake.

### Definitions

This study aims to determine the safety and efficacy of the use of TONGEE DJBL in the treatment of patients with weight recurrence in sleeve gastric surgery. Technical success is defined as the successful placement of the stent into the duodenum and its successful deployment. Adverse events assessed included bleeding, abdominal pain, and stent displacement. Bleeding was defined as any event requiring endoscopic intervention, radiologic intervention, red blood cell transfusion, and/or hospitalization to achieve hemostasis. The severity of adverse events was determined according to the AGREE (Adverse Gastrointestinal and Respiratory Events) classification system. Operative time is defined as the number of minutes from endoscopic entry to successful stent placement.

The main outcome of this study is to evaluate the percentage of total weight loss (TWL) and excess weight loss (EWL = weight loss/overweight). A BMI of 24 kg/m ^2^ was used to define the ideal weight for the overweight.

### Statistical Analysis

Continuous variables are represented as means using SD, while categorical variables are represented as counts and percentages. All tests were double tailed, with a significance level of alpha = 0.05, and analyzed and performed using SPSS 26.0 software (IBM Corporation, Armonk, NY, USA). One-way ANOVA was used to compare whether the differences in means between two or more samples are significant. Statistical charts were prepared by GraphPad Prism 10.0.

## Results

**Fig. 3:**
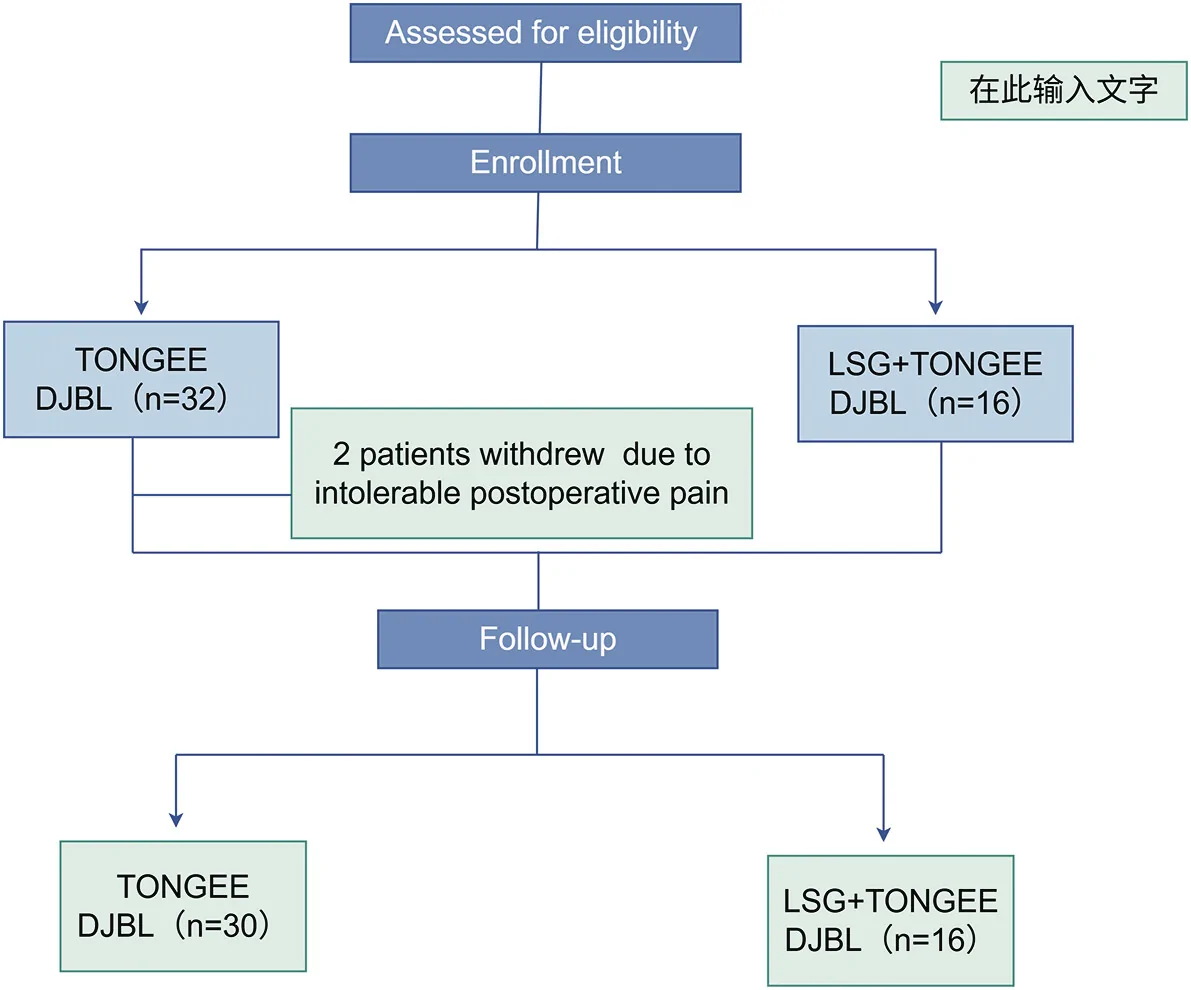
Study flow diagram.

During the study period, a total of 48 patents were treated using TONGEE DJBL. All patients were successfully implanted. Two patients withdrew from the study due to intolerable postoperative pain after stent placement (both from the simple obesity group), and a total of 46 patients have completed a 3-month follow-up. Table 1 shows the specific basic information of the two groups, and it can be seen that there is no statistical difference in the basic information between the two groups of patients ( Fig. 3 ).

**Table 1:** The characteristic information of two groups.

	TONGEE DJBL	LSG + TONGEE DJBL	*P* -value
	( *n* = 30)	( *n* = 16)	
Age (mean ± SD)	36.97 ± 7.92	41.56 ± 10.74	0.105
Gender			0.493
Male	10	3	
Female	20	13	
Weight (kg) (mean ± SD)	96.53 ± 21.54	88.22 ± 16.12	0.183
BMI (kg/m ^2^ ) (mean ± SD)	33.16 ± 5.40	31.48 ± 3.07	0.258

In all, 6 out of the 46 patients reported experiencing abdominal pain after implantation surgery (four in the TONGEE DJBL group and two in the LSG + TONGEE DJBL group), and the pain was relieved within 2 weeks.

Table 2 shows the weight loss effects of the two groups after 3 months. Although the average EWL of the TONGEE DJBL group is higher than that of the LSG + TONGEE DJBL group, there is no statistical difference in total weight loss percentage (TWL%) and excess weight loss percentage (EWL%) between the two groups.

**Table 2:** The weight loss effect of the two groups after 3 months of stent implantation.

	TONGEE DJBL	LSG + TONGEE DJBL	*P* -value
	( *n* = 30)	( *n* = 16)	
TWL% (mean ± SD)	10.98 ± 5.96 [8.84, 13.11]	10.36 ± 3.77 [8.51, 12.21]	0.710
EWL% (mean ± SD)	50.87 ± 37.28 [37.54, 64.22]	50.37 ± 29.16 [36.09, 64.67]	0.963

**Fig. 4:**
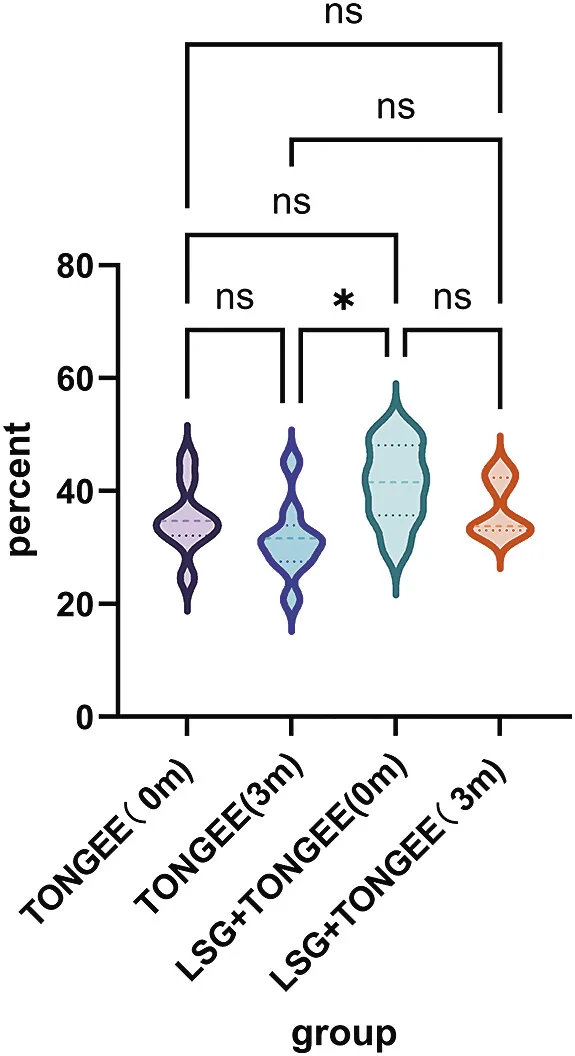
The changes in body fat percentage before and 3 months after surgery for each group.

Fig. 4 compares the changes in body fat percentage between the two groups before surgery (0m) and 3 months after surgery (3m). It can be seen from the figure that the body fat percentage decreased in all groups after surgery (TONGEE DJBL group decreased by 4.43%; LSG + TONGEE DJBL group decreased by 5.36%). From the graph, it can be seen that the body fat percentage of the TONGEE(3m) group is significantly lower than in the preoperative LSG + TONGEE group.

**Fig. 5:**
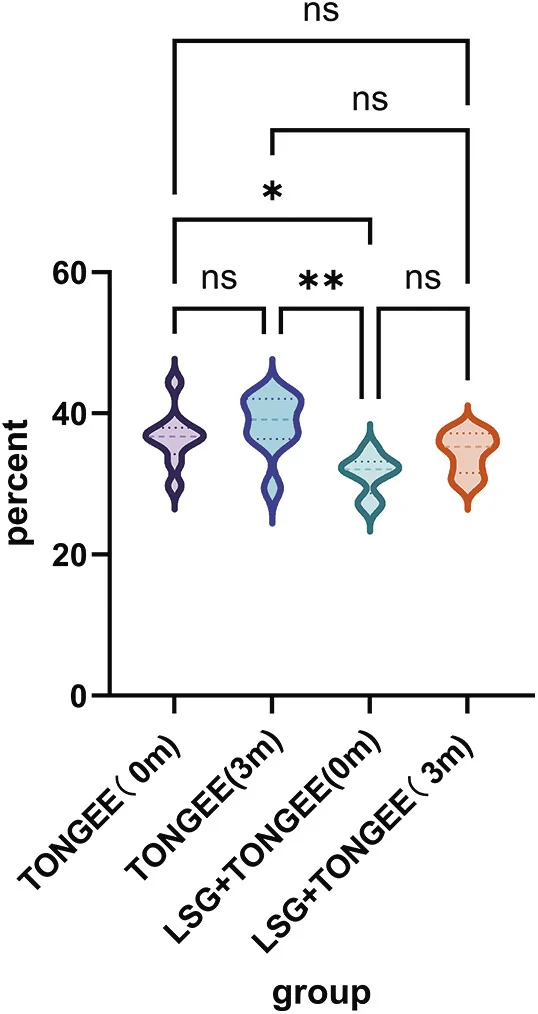
The changes in muscle rate before and 3 months after surgery for each group.

Fig. 5 shows the changes in muscle rate of the two groups of patients before surgery (0m) and 3 months after surgery (3m). From the graph, it can be seen that the muscle mass of the preoperative LSG + TONGEE group was significantly lower than that of the TONGEE DJBL (0m and 3m) groups. Three months after surgery, the average muscle mass of both groups of patients increased by 2.14% and 3.30%, respectively. Three months postoperatively, the muscle mass of patients in the TONGEE group and the LSG TONGEE group was significantly higher than before surgery.

## Discussion

The aim of this study is to determine the efficacy and safety of using the TONGEE gastric bypass stent for the treatment of weight recurrence in patients with LSG. The clinical success rate of the technology is 100%, with no serious adverse events.

Our center reported the world’s first case of applying a gastric bypass stent to a patient who regained weight after LSG in January 2024 in the *Asian Journal of Surgery* . This article further compared the weight loss effects between the TONGEE DJBL group and the LSG + TONGEE DJBL group. Under the condition of matching basic conditions, there was no significant statistical difference in postoperative complications and weight loss effects (TWL and EWL) between the two groups 3 months after surgery. Both groups of patients experienced a decrease in body fat percentage and an increase in muscle percentage 3 months after surgery, indicating that the weight loss effect of gastric bypass stents was mainly due to fat reduction. However, there was no statistically significant difference in the improvement of body fat percentage between the two groups after surgery. Notably, 3 months after surgery, patients in both the TONGEE group and the LSG TONGEE group experienced a significant increase in muscle mass.

A newly published meta-analysis included 30 studies involving 1751 patients with a stent placement time of 12 months. The average EWL of patients was 41.3% (95% CI 33.4%, 49.2%), and the average TWL was 13.1% (95% CI 10.1%, 16.0%). The weight loss effect was better than our study, which may be due to different stent-placement times. The early removal rate of the stent was 19%, and the incidence of serious adverse events was 17%, including device displacement (6%), gastrointestinal bleeding (4%), device obstruction (4%), and liver abscess (2%), which were significantly higher than our study. This may be due to the improvement of stent material and different implantation time. 8

The latest multicenter clinical study included 1022 patients from 33 centers in 10 countries, with a placement period of 12 months. The results showed that the average TWL of the patients was 11.1%, and they all had benefits in blood glucose and cardiovascular diseases. The study suggests that shortening the stent placement time can reduce the incidence of adverse reactions. 10

As an endoscopic weight loss therapy, duodenal jejunal tube insertion has been applied in clinical practice for more than 10 years. EndoBarrier first reported the treatment of obesity and type 2 diabetes in 2007. 12 The results of a multicenter randomized controlled trial involving 170 type 2 diabetes and obese adults with poor glycemic control showed that, compared with intensive drug treatment, the weight of EndoBarrier group patients was significantly reduced at 12 months, and blood pressure, serum total cholesterol, and ALT and AST were significantly reduced, but there was no statistically significant difference in the reduction of glycosylated hemoglobin. 13 14

Currently, postoperative weight gain after LSG is a difficult problem in the field of weight loss and a research hotspot. Research shows that the 10-year corrective surgery rate for LSG can reach 33%. 15 There is currently no unified standard for the selection of corrective surgery for postoperative weight gain after LSG, but the exploration direction of its surgical procedures mainly focuses on retrospective sleeve gastrectomy (ReSG), RYGB, one anastomosis gastric bypass (OAGB), and sleeve gastrectomy plus (SG Plus) surgery. 16 Compared to surgical procedures, endoscopic treatment has the advantages of less trauma, lower risk, and lower cost. The data from this study suggest that LSG + TONGEE DJBL has the same weight loss effect as TONGEE DJBL and can be used as an option for patients who regain weight after LSG surgery.

This study has the following shortcomings: a small sample size and short follow-up time. Subsequent studies should continue to increase the number of research cases, extend follow-up time, and provide further research data.

In summary, the TONGEE gastric bypass stent can be used as a treatment option for patients who have regained weight after LSG surgery.

## Data Availability

The datasets generated during and/or analyzed during the current study are available from the corresponding author on reasonable request.
